# Assessment of aging of anaerobic digester paddle-mixer material: experimental studies and long-term numerical simulation

**DOI:** 10.1007/s00449-023-02862-9

**Published:** 2023-03-22

**Authors:** Spyridon Psarras, Thomas Zaragkas, Dimitris Pegkos, Polyxeni Dimoka, Alexandros Eftaxias, Panagiotis Charitidis, Vasileios Diamantis, Alexandros Aivasidis, Vasileios Kostopoulos

**Affiliations:** 1 Department of Mechanical Engineering and Aeronautics, University of Patras, 26500 Patras, Greece; 2 Department of Environmental Engineering, Democritus University of Thrace, 67100 Xanthi, Greece

**Keywords:** Agitation system, Anaerobic digestion, Biogas, Thermoplastic, Composite materials, Finite element modeling

## Abstract

In this study, experimental tests and numerical simulations (Abaqus) were performed to examine the durability of four impeller materials [steel, polyethylene, polypropylene and glass fiber reinforced polymer (GFRP)] in an anaerobic digester environment. Specimens of these materials were prepared and immersed in a bath containing anaerobic digester liquor while operated at 40 °C for a period of 8 months. Periodically (2, 4, 6 and 8 months) sample specimens were removed from the bath and the tensile strength and elastic modulus were determined. As expected, thermoplastic materials and especially GFRP exceeded higher absorption of moisture than steel, although aging effect on steel was more pronounced due to corrosion, as evidenced by SEM imaging. The results demonstrate that polyethylene was not acceptable as construction material for anaerobic digester paddle mixer. On the contrary steel, GFRP and PP remained highly unaffected with a negligible increase of the maximum stress, 1.6%, 0.9% and 3.0%, respectively.

## Introduction

Biogas and its upgraded form biomethane, a substitute to fossil natural gas, can accelerate Europe’s green energy transition and contribute to energy security. According to the European Biogas Association (EBA), at EU level there are 19,000 anaerobic digestion facilities generating around 18 bcm of methane [1]. The number of anaerobic digestion facilities is expected to double by 2030 if the REPower EU target of producing 36 bcm of biomethane is achieved. Clearly, to reach this goal, we need financial incentives, new legislation and social awareness measures [2]. Valorization of novel substrates (e.g., lignocellulosic biomass, renewable hydrogen) combined with new technologies and business models (decentralized biogas production and upgrading), will clearly boost environmental technology market, over the next decades.

Anaerobic digestion facilities are designed to optimize contact between the incoming substrate and the digester biomass. Therefore, agitation systems are crucial components that provide homogenous dispersion in tank vessels, maximize mass and heat transfer thus ensure high process efficiency [3]. Several types of mixers-impellers are currently used for different operations in process industries [4–6]. The most common type of mixing systems in biogas production facilities are high-shear propellers and low shear paddles [7]. Generally, slow mixing is preferred due to lower power consumption and shear applied [8, 9].

In an anaerobic digestion tank the impellers/paddles are submerged into the slurry [9]. Because the biogas plant operates continuously over time, reliability is a critical issue because equipment failure results in production shutdown. As a consequence, the economic feasibility and investment depreciation are adversely affected [10]. Moreover, digester mixed liquor characteristics, such as fiber content and rheology, as well as digester type, are critical factors for agitator selection and design [8, 11, 12]. Material degradation, as well as preexisting notches or flaws, initiate cracks that grow and lead to fractures under the combined stresses created by fluctuating forces during operation. During mixing, the agitation torque is increased as exerting load on agitator blades [12]. The magnitude of torque depends on the blade design, the rotational speed, the density of slurry as well as the type of mixer [12–14]. Previous studies investigated the corrosivity of construction materials in wastewater treatment plants [15–18]. Among different types of damages (erosion, abrasion and corrosion) the most critical is erosion [19–21]. Cast iron, steel and stainless steels are widely used in the construction of wastewater treatment plant components [22]. Ductile materials (steels) are significantly influenced by slurry erosion because of their microstructure. To protect carbon steels, polymeric coatings such as polyethylene is often applied [23, 24].

However, aiming to reduce the maintenance costs associated with corrosion, alternative reinforcing materials, such as Fiber Reinforced Polymers (FRP), have been investigated over the past decades [25, 26]. Composite materials like Glass Fiber Reinforced Polymer (GFRP) composites provide superior mechanical properties [27, 28]. Several authors recognized that aggressive environments can be detrimental to the matrix and the fibers [29, 30], resulting in the deterioration of mechanical properties such as elastic modulus and strength with increasing exposure time [31, 32].

Thermoplastic materials such as polyethylene and polypropylene are widely used in various industries. The degradation of thermoplastic materials by different solvents have been reported recently, such as toluene/ methanol mixture [33], a water solution of sulfuric acid and crude oil [34], isooctane/toluene mixture [35], cycloalkanes and aromatic hydrocarbon [36], diesel and biodiesel fuel [37]. According to Refs. [33–37], the PE swelling capacity depends on the PE specimen preparation. Mobility of PE macromolecules as a function of temperature was also reported [35, 37].

The aim of this study was to evaluate the effect of GFRP, PE, PP and steel aging when immersed into an anaerobic digester environment. For this reason, an aging test campaign was designed using raw anaerobic digester liquor and operated at 40 °C for a period of 8 months. Specimens of different materials were periodically removed from the digester liquor and tested in tension. The experimental data obtained were used to simulate long-term material performance with the aid of finite element models. In addition, the aging propagation was monitored through SEM imaging and the failure mechanisms involved were studied in detail for each material. Using the Fickian law for moisture uptake the diffusion properties for all materials were derived and compared with diffusion analyses performed by the ABAQUS heat transfer solver.

## Materials and methods

### Anaerobic digester liquor

**Fig. 1:**
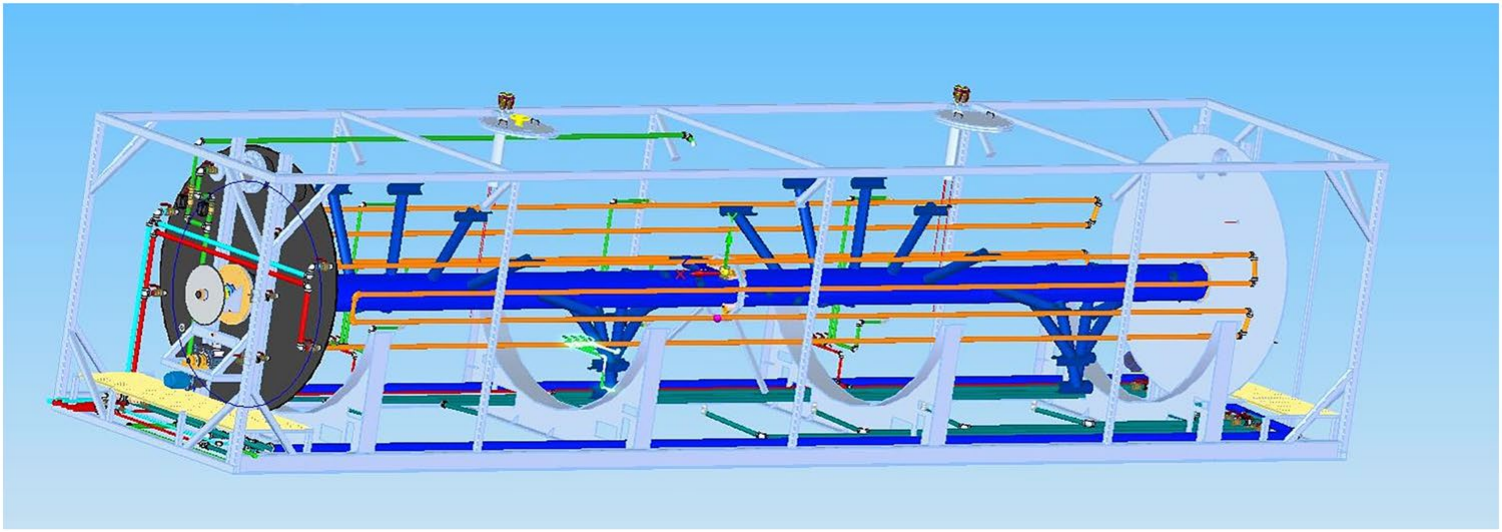
Schematic representation of the anaerobic plug-flow reactor used for the study, showing the installed horizontal paddle-mixing system

The anaerobic digester liquor used in “aging studies” was obtained from an anaerobic plug-flow reactor with 50 m^3^ working volume (Fig. 1). The anaerobic digester was operated under mesophilic conditions (38–39 °C) while treating mainly screened dairy manure. During the operation period, samples were obtained to assess the composition of anaerobic digester liquor. The analytical methods included determination of the electrical conductivity, pH, ammonia–nitrogen, total suspended solids and volatile suspended solids, according to the Standard Methods for the Examination of Water and Wastewater [38]. Volatile fatty acids concentrations were determined according to Diamantis et al. [39].

### Material and tensile test conditions

**Fig. 2:**
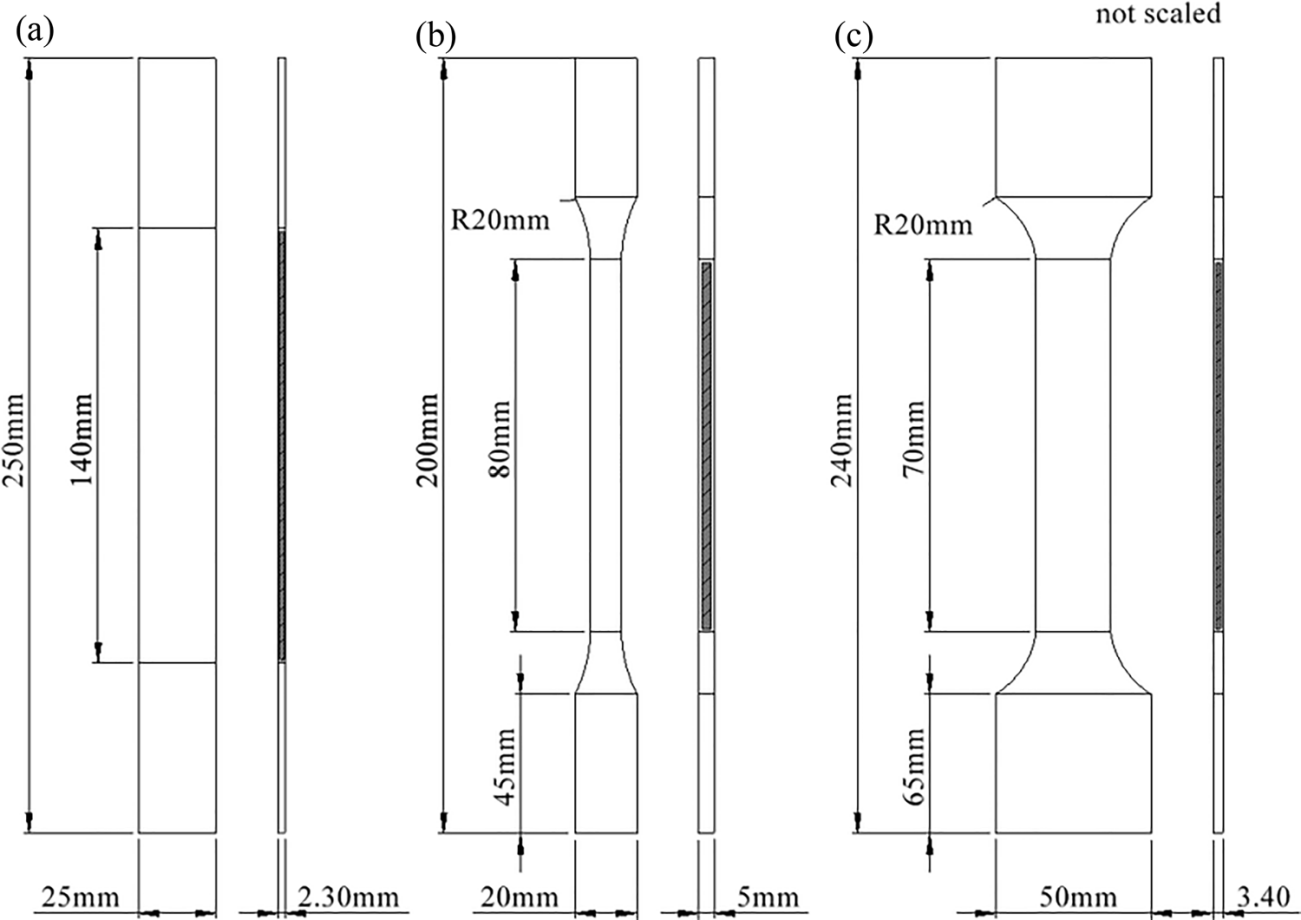
Dimensions of specimens of **a** GFRP, **b** thermoplastic materials and **c** steel (St-37)

The testing configurations were based on the ASTM standards E345-16 and ASTM D638-14 for metal and plastic materials, respectively. The specimen dimensions per type of material are shown in Fig. 2.

**Fig. 3:**
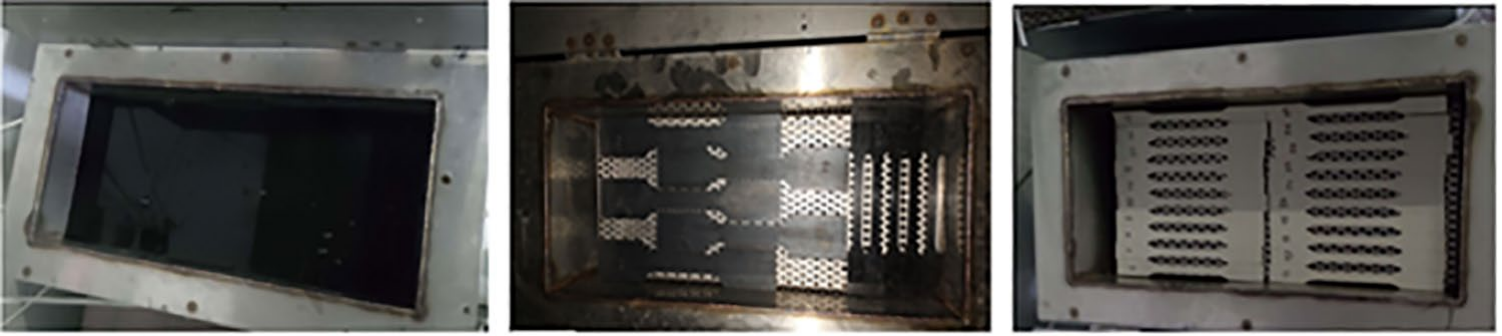
Direct immersion of specimens in metal bath containing anaerobic digester liquor

Four different materials were used to model the specimens. The first specimen material was steel (st-37) with thickness of 3.40 mm. Alternative materials such as glass fiber reinforced polymer composite as well as thermoplastic materials (polyethylene, polypropylene) were also used. Thermoplastic materials and steel were kindly provided by Stemplast Company (Greece) and Chryssafidis company (Greece), respectively. Moreover, glass fiber reinforced composite material (Sigratex GE 8903-280-37S) was also analyzed. Before uniaxial testing, the specimens were immersed in the anaerobic digester liquor at 40 °C (Fig. 3). The specimens were removed from the aging solution periodically at 2, 4, 6 and 8 months. Seven specimens for each case (140 specimens were manufactured) were tested on two different Universal testing machines (Instron 8872 and Instron 8802). The specimens were tested up to failure, while a displacement rate of 1 mm/min was selected. Tensile strength and Young’s modulus were determined by the tension tests.

### Finite element model

For the modelization of paddler mixer, embedded 8-node solid elements were used to evaluate the impeller strength to calculate the effects of steady loading conditions on the structure in ABAQUS. Fine mesh was used at the vicinity of the critical points, while an equivalent force at the tip of the impeller was applied. Generally, static analysis was selected since it provides whether a component withstands the maximum stresses. Such failure analysis was necessary.

Both experimental and theoretical analyses were done to maximize the performance of the impeller by material optimization. It should be noted that Poisson’s ratio of the thermoplastic materials and steel was not determined experimentally. On the other hand, for the case of GFRP the Poisson ratio was considered constant for all aging periods. The following assumptions were made:

For Steel, PP and PE.

- Poisson's ratio was not found experimentally, but from the literature as transverse deformations were not measured in tensile experiments.
- The measure of elasticity was quantified through exponential forecasting, while the maximum tensile stress and the maximum deformation were provided by a linear law.
- Density and Poisson ratio were considered constant over time.
- In the definition of plastic behavior in ABAQUS, the curve ${\sigma }_{\mathrm{true}}=f({\varepsilon }_{\mathrm{plastic}})$ was used for the reference experiment (0 months) and in the following months a corresponding decrease in the stress values and an increase in the deformation values were considered, based on the prediction of maximum stress and strain.

For GFRP

- Tensile tests with aging were performed in the main direction 1. The reduction of the other elastic constants was done in the same way for the same reasons.
- Poisson ratios here were subjected to reduction, relative to other materials that were considered stable over time.

## Results and discussion

### Physicochemical properties of anaerobic digester liquor

**Fig. 4:**
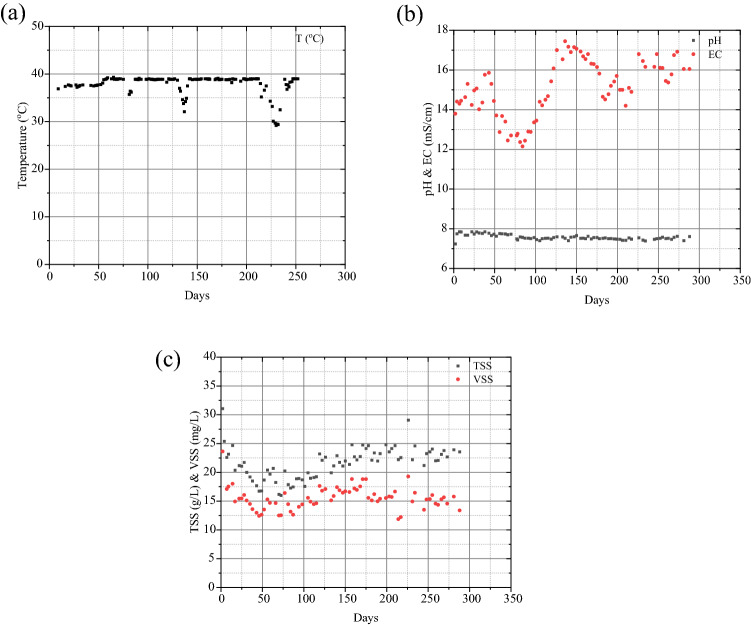
Physicochemical properties of the anaerobic digester liquor during the study period: **a** temperature, **b** pH and electrical conductivity, **c** total and volatile suspended solids

Figure 4 shows the anaerobic digester liquor temperature during the study period. The latter remained around 38–39 °C with some exceptions due to temporal failure of the digester heating system. The pH inside the anaerobic digester remained constant at 7.4–7.6 which is considered within the optimum range for methanogenic bacteria [3]. The electrical conductivity varied between 12 and 17 mS/cm and it was mainly affected by the salinity of the incoming wastes. Similarly, the concentrations of total and volatile suspended solids inside the anaerobic digester were between 17–25 and 12–18 g/L, respectively.

**Fig. 5:**
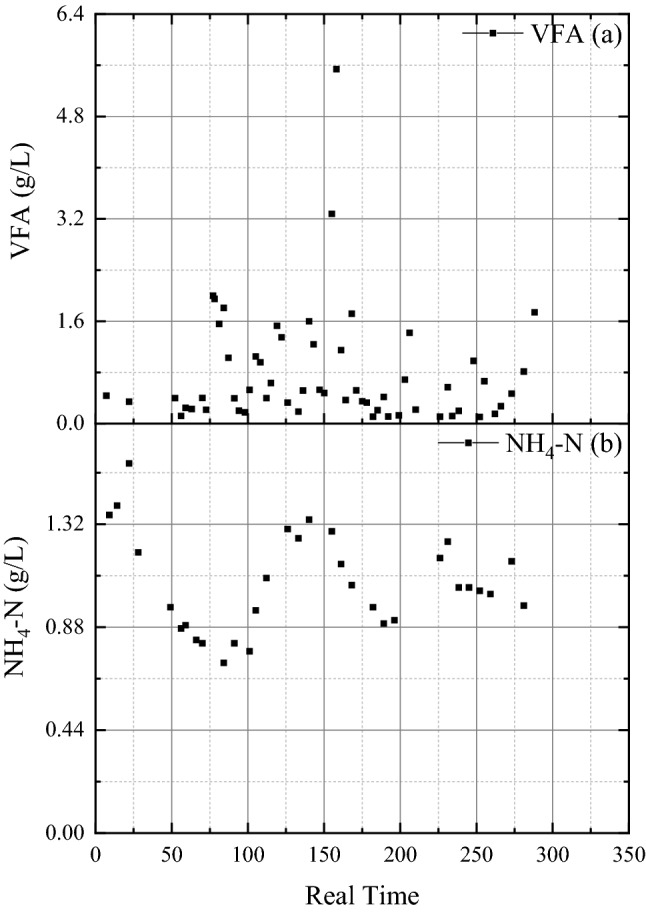
Concentrations of **a** ammonia nitrogen and **b** volatile fatty acids during anaerobic digester operation

As seen from Fig. 5, ammonia nitrogen concentration varied between 800 and 1400 mg/L, similar to the electrical conductivity, while the volatile fatty acids concentrations remained below 1500 mg/L in most of the measurements. Considering the above, the composition of the medium used for the “aging studies” is shown in Table 1.

**Table 1:** Physicochemical properties of anaerobic digester liquor used in aging studies

Parameter	Value
Ph	7.58 ± 0.13
EC (mS/cm)	15.1 ± 1.4
TSS (g/L)	21.6 ± 2.8
VSS (g/L)	15.5 ± 1.9
PO_4_-P (mg/L)	62.0 ± 14.0
NH_4_-N (mg/L)	1070 ± 210
VFA (mg/L)	790 ± 90

### Material degradation and durability

The main purpose of the experiments was to investigate the degradation of materials, under the conditions prevailing inside an anaerobic digester. As seen from Table 2, between different thermoplastic materials, the thickness of the polyethylene specimens decreased by 9.82%.

**Table 2:** Dimensions of the specimens before and after aging studies using anaerobic digester liquor

Materials	Ref. specimens		Aging period 8 months	
	Width (mm)	Thickness (mm)	Width (mm)	Thickness (mm)
Steel-37	23.9	3.92	24.0	3.87
PP	10.0	5.10	10.0	5.10
PE	10.0	4.81	10.0	4.38
GFRP	24.8	2.30	24.8	2.31

On the other hand, minor changes were observed on steel specimens where the width increased by 0.24% and the thickness decreased by 0.77%. Therefore, to understand the differences in the dimensions of the specimens, tensile strength was selected to evaluate the performance of specimens subjected to an anaerobic digester environment.

According to Table 3, the tensile strength of steel specimens decreased with increasing aging time. The reduction of the tensile strength (5.96%) was attributed to the chemical composition of the digester liquor (ammonia, salinity and organic acids) which increased the corrosion rate. Furthermore, knowing the stress–strain curves, the Modulus of Elasticity was predicted. Table 4 shows Youngs’ modulus results for reference specimens and after 2, 4, 6 and 8 months of aging, respectively.

**Table 3:** Tensile strength results of the specimens as a function of the aging period

		Aging period				
		Reference	2	4	6	8
Steel (St 37)	Tensile strength (MPa)	405	404	406	395	383
	Standard deviation	3.4	2.6	19.1	16.1	5.7
Polypropylene	Tensile strength (MPa)	35	35	33.9	33.4	36.8
	Standard deviation	0.2	0.3	0.1	0	4.5
Polyethylene	Tensile strength (MPa)	25.2	25.5	24.4	231	24.9
	Standard deviation	0.6	0.3	0.5	0.6	0.6
GFRP	Tensile strength (MPa)	406	330	348	345	316
	Standard deviation	0.05	0.03	0.01	0.03	0.01

**Table 4:** Specimen’s modulus of elasticity as a function of the aging period

Modulus of elasticity (GPa)				
Months	Steel	Polypropylene	Polyethylene	GFRP
0	204	1.84	1.26	22.8
2	202	1.58	1.09	22.3
4	199	1.67	1.06	21.8
6	197	1.59	1.01	22.6
8	194	1.52	0.93	22.1

### Aging mechanisms

**Fig. 6:**
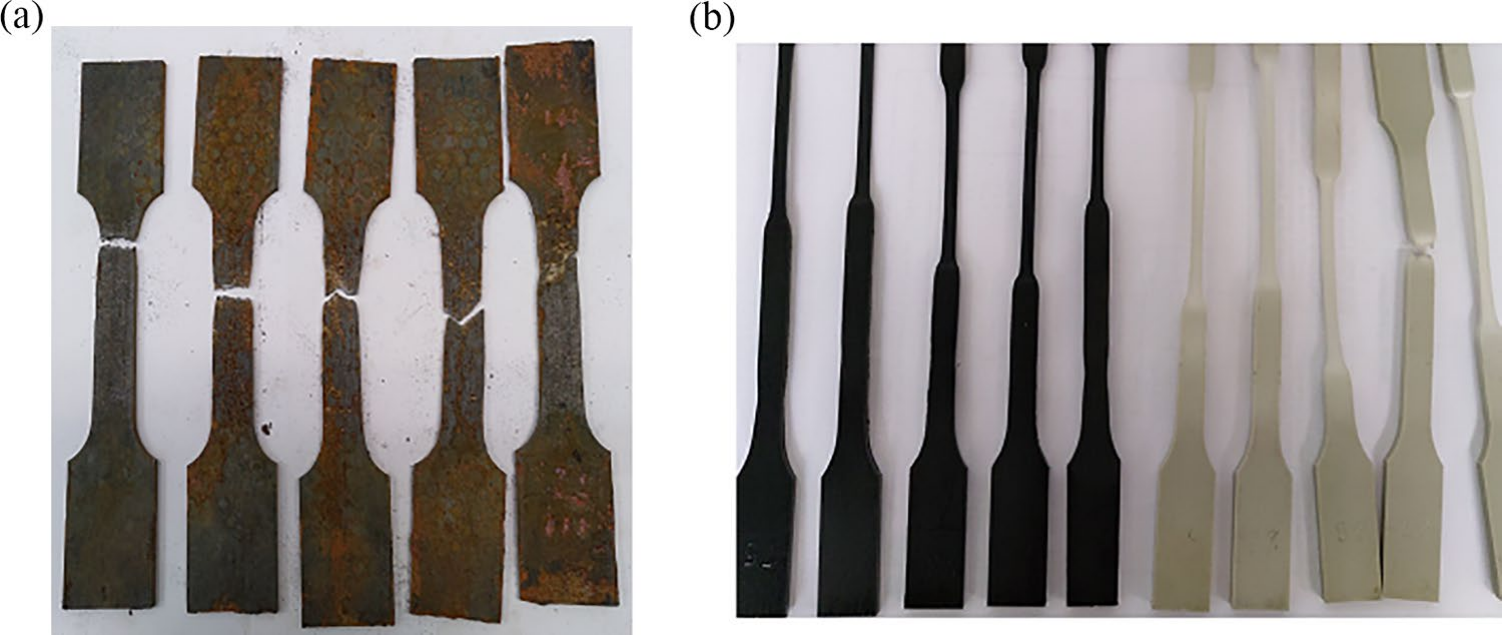
**a** Corrosion of St 37 specimen because of hygrothermal aging. **b** Plastic deformation of thermoplastic materials

After 8 months aging the materials’ properties have been altered because of hygrothermal failure mechanisms. The Steel specimens were severely affected due to corrosion (see Fig. 6).

**Fig. 7:**
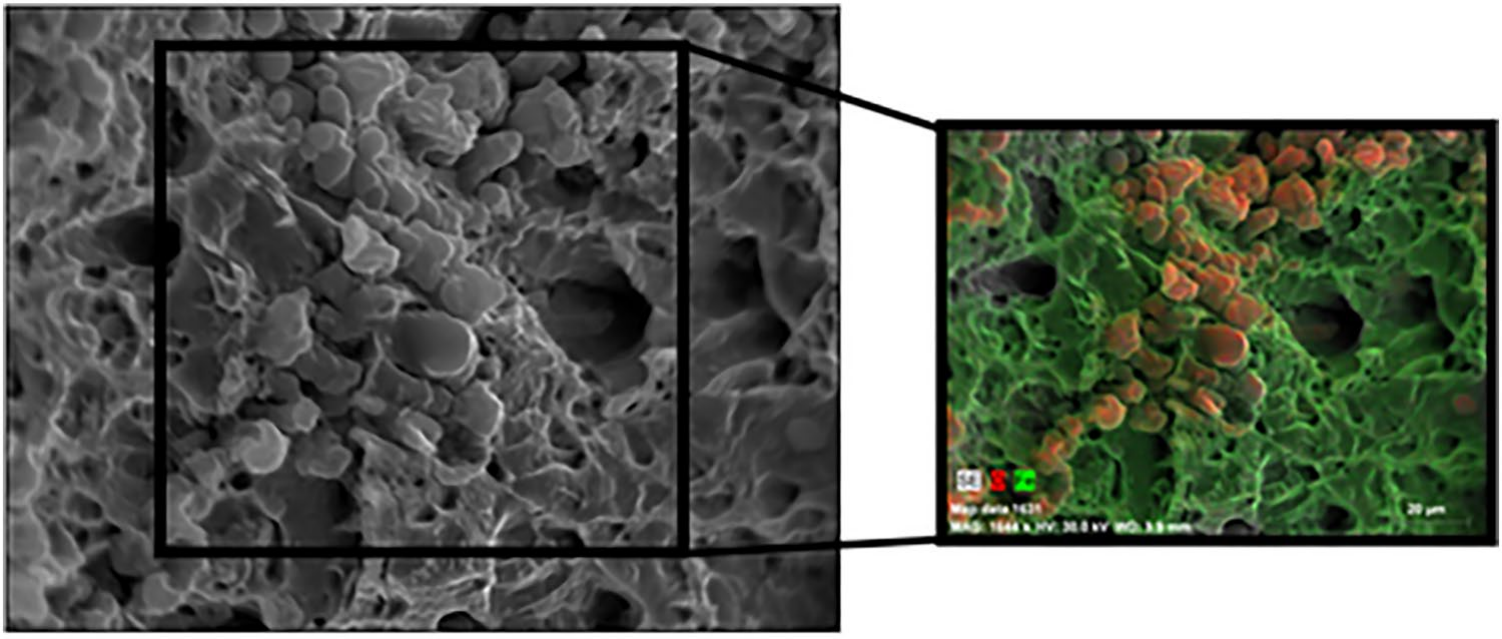
EDX analysis of steel specimen after 6 months of aging

**Fig. 8:**
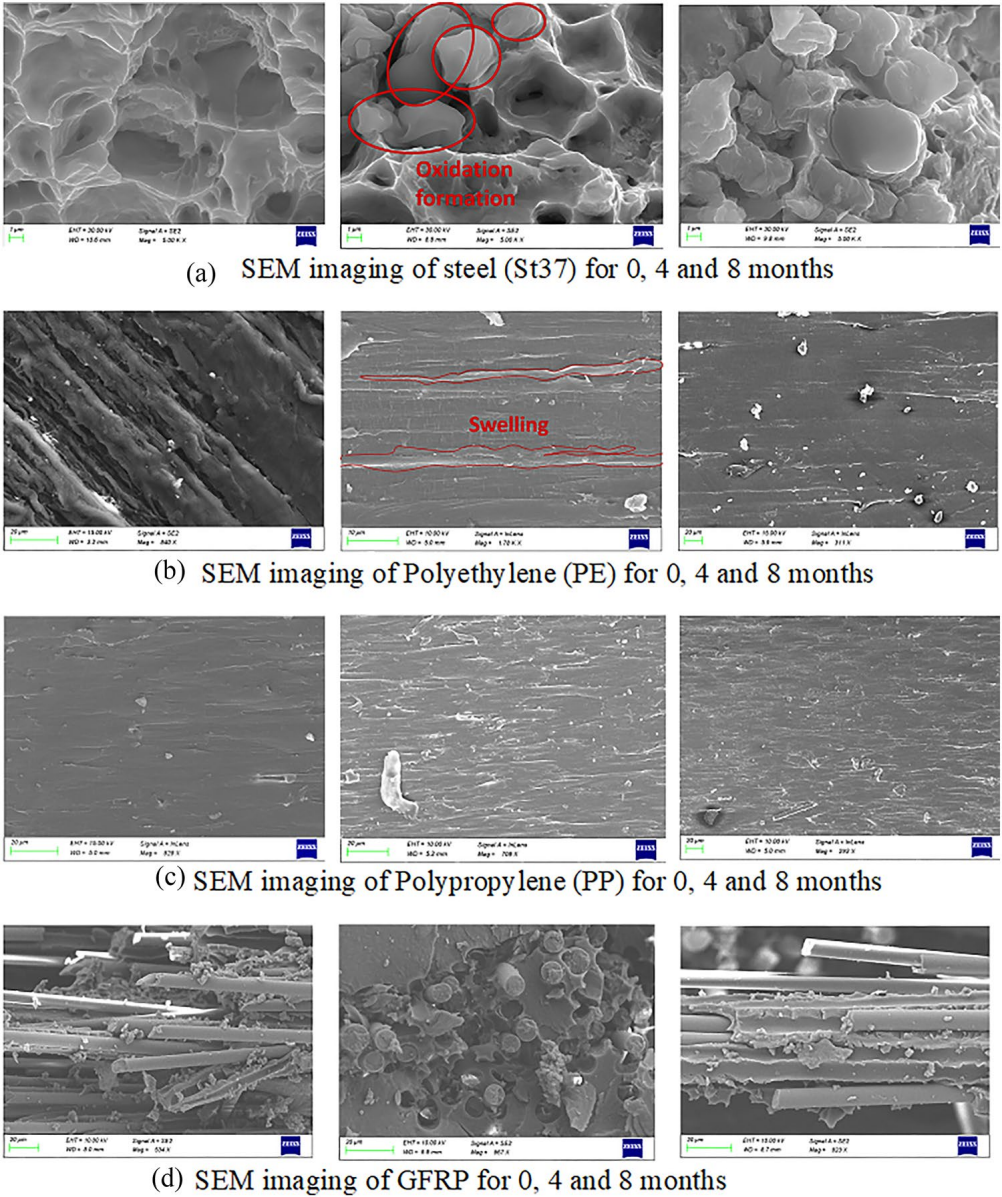
SEM imaging of **a** St37, **b** PE, **c** PP and **d** GFRP for 0, 4 and 8 months

Material degradation as a function of aging time and the effect on failure mechanisms for different type of specimens was recorded through SEM imaging (Fig. 7). Steel samples showed, within the first two months, a high degree of oxidation as evidenced by the energy-dispersive X-ray analysis (EDX) (Fig. 8). Since the thermoplastic coupons had not been fractured rather than plastically deformed the SEM images were obtained from the side of the samples. For GFRP samples, fiber-matrix debonding was recorded after 8 months of aging, however, glass fibers (ceramic material) were not significantly affected, while moisture penetrated between the fibers and the matrix. Swelling of thermoplastics and GFRP was obvious only from dimensional changes and SEM imaging, together with matrix cracking, debonding and delaminations of the GFRP.

### Long-term predictions

**Fig. 9:**
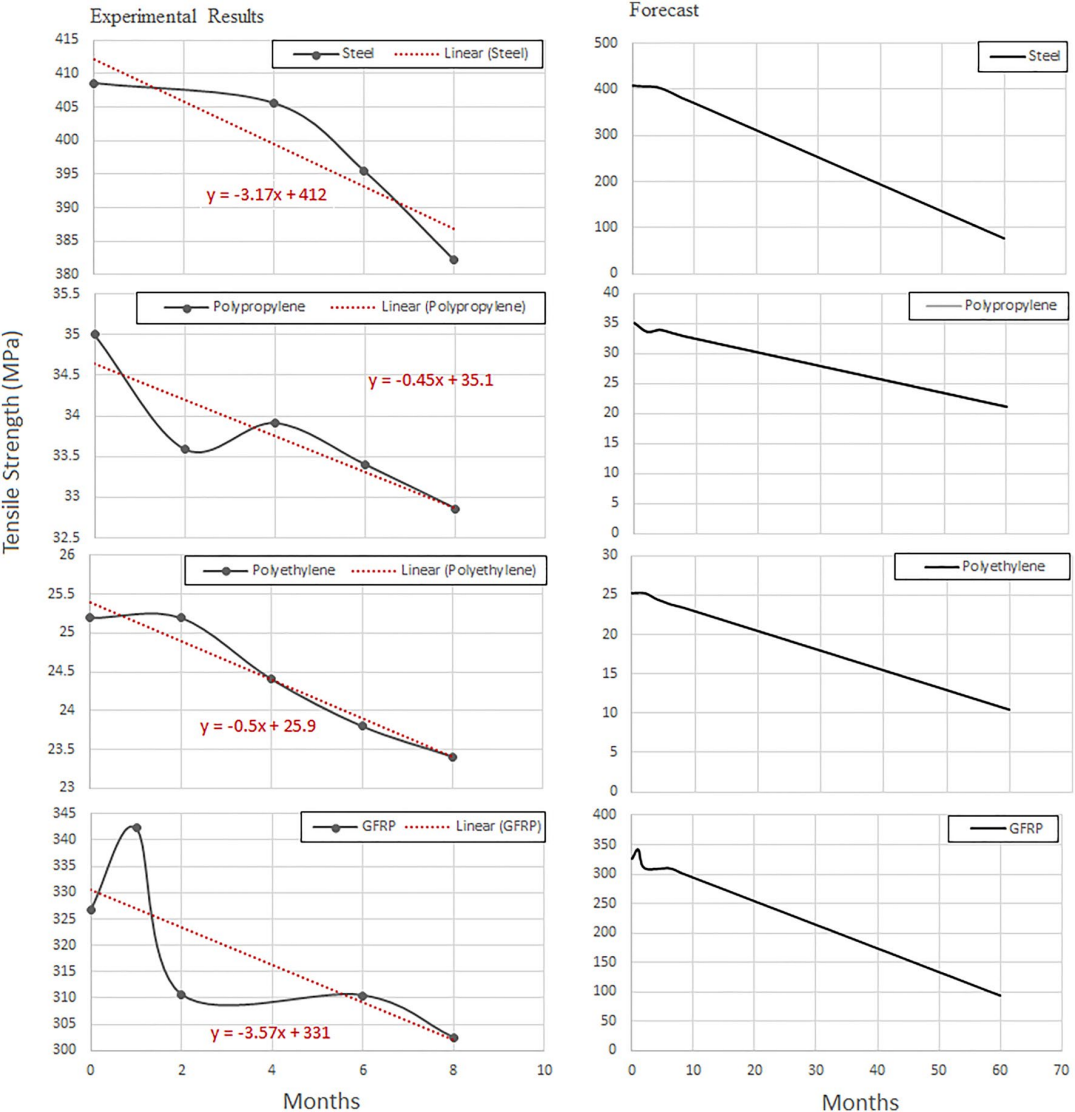
Long-term prediction of tensile strength (MPa) of polyethylene, polypropylene, steel (St-37) and GFRP

**Fig. 10:**
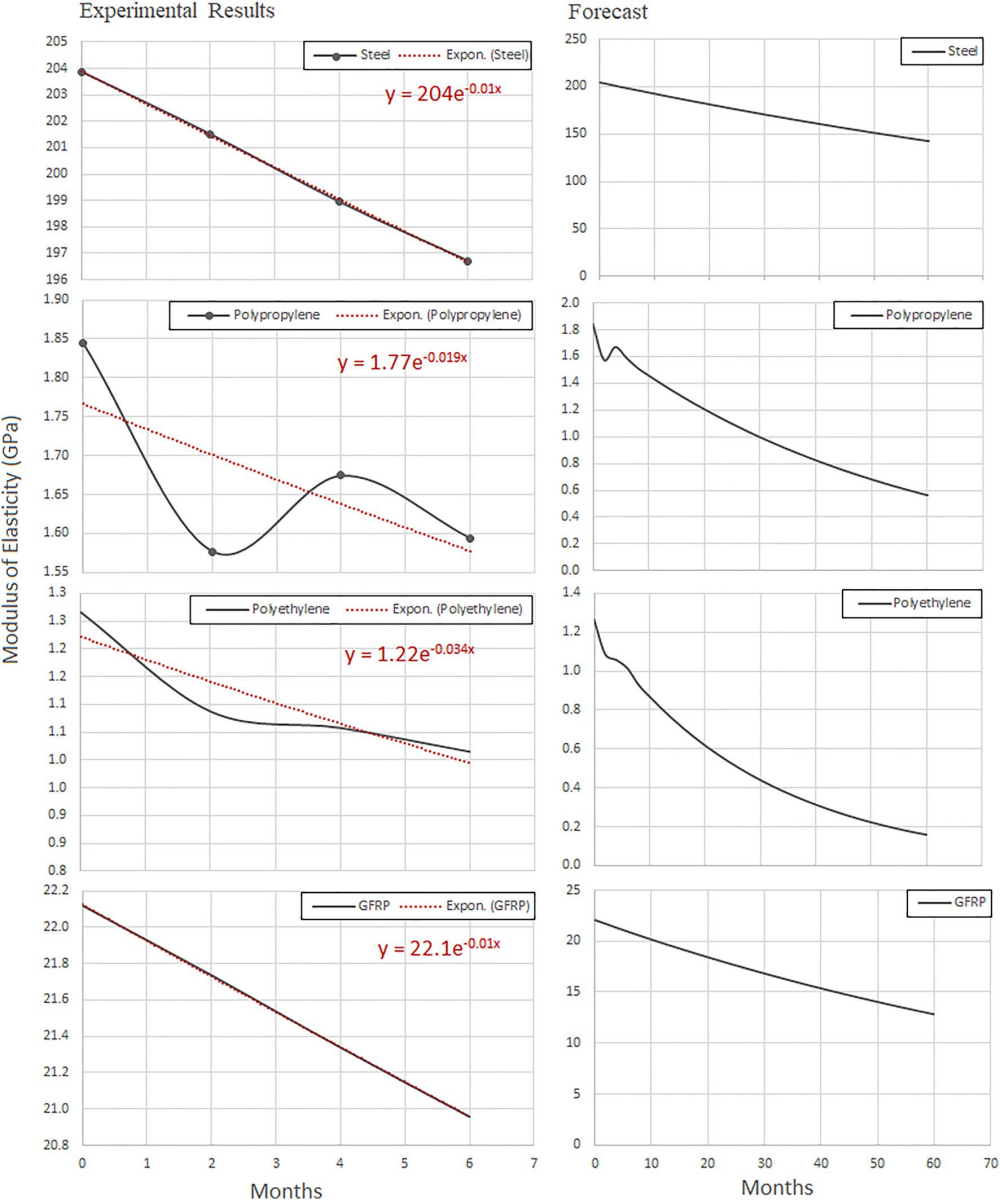
Long-term prediction of Young’s modulus (GPa) of polyethylene, polypropylene steel (St-37) and GFRP

Figures 9 and 10 show the long-term predictions of tensile strength and modulus of elasticity for polyethylene, polypropylene, steel and GFRP, respectively. The degree of degradation of the modulus of elasticity after 5 years’ time, was determined by a nonlinear regression model [40–42]. Figures 9 and 10 show that polyethylene was significantly affected by the applied conditions. The tensile strength and modulus of elasticity decreased by more than 141% and 695%, respectively, with increasing aging time.

Generally, fiber reinforced polymer composites are based on the high strength fibers in a matrix that provides favorable bonding. GFRP was in a prepreg form; 10 layers of 0° resulted in a layer thickness of 2.3 mm. The mechanical properties were *E*_1_ = 22.8GPa, *E*_1_ = *E*_2_ = 7.5GPa, *G*_1_ = *G*_2_ = 3.4GPa, *v*_12_ = *v*_13_ = 0.13, *v*_23_ = 0.17. After 6 months of aging period, mechanical properties decreased by 0.83% (*E*_1_ = 21.6GPa). An exponential function was used [*E*11 = 22.1e^−0.009t^ (GPa)], to predict potential reduction of the modulus of elasticity in one-direction. Table 3 verifies that with increasing time period, the mechanical properties deteriorated.

## Finite element analysis results

### Diffusion analysis

In this section, a comparison was made between experimental and numerical results for different sample moisture uptake. Diffusion analysis was performed, and the experimental findings were compared with the results of ABAQUS heat transfer model. The values of diffusion obtained experimentally for all samples examined are presented in Table 1. The 8-node linear heat transfer brick (DC3D8) elements were used, and mass concentration was applied to all outer surfaces of the specimens (Table 5).

**Table 5:** Diffusion property derived experimentally for different sample specimens

$\mathrm{Material}$	$\mathrm{D }\times 1{0}^{-7} ({\mathrm{mm}}^{2}/\mathrm{s})$
PP	4.62
PE	3.85
Steel	0.37
GFRP	8.71 (fiber direction), 0.43 (transverse directions)

**Fig. 11:**
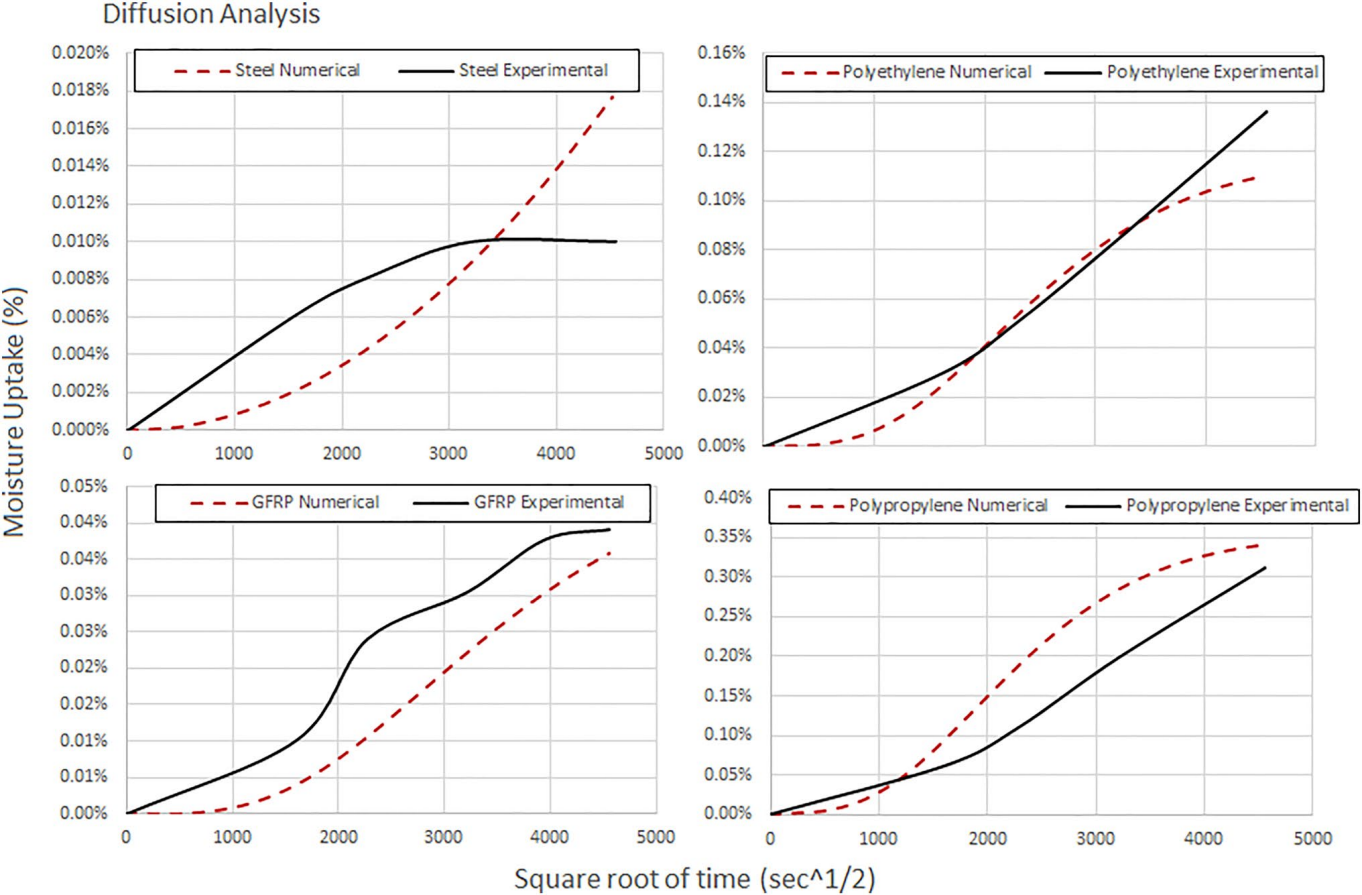
Comparison curves between experimental and numerical results for moisture uptake

**Fig. 12:**
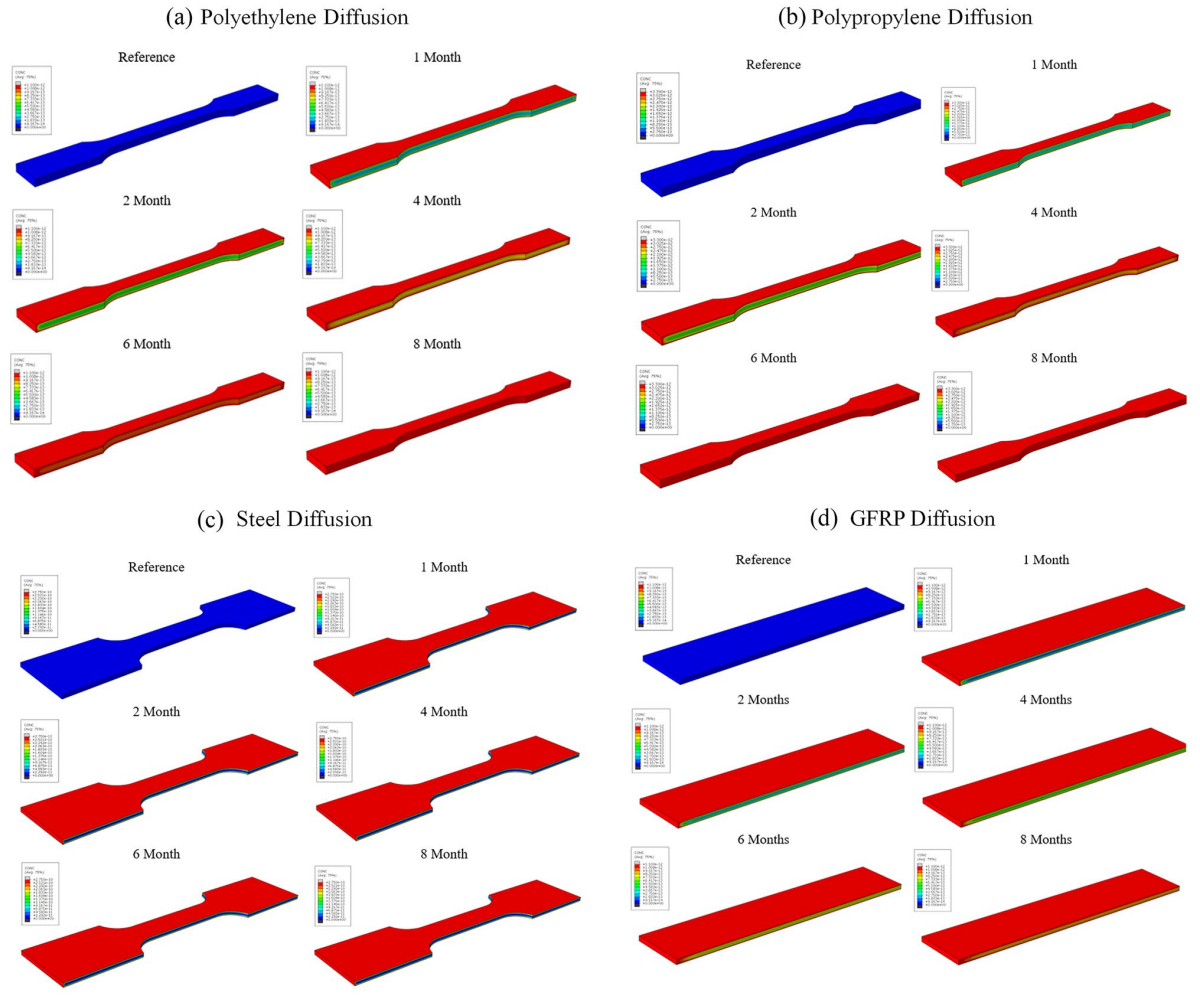
FE predictions of diffusion for **a** polyethylene, **b** polypropylene, **c** steel and **d** GFRP for time period 0–8 months

As evidenced by the data in Fig. 11, thermoplastics (PP, PE) seem to have fulfilled the moisture uptake though steel and GFRP not. The numerical result of steel seems to overpredict the uptake. All FE predictions of diffusion for the materials of this study are presented in Fig. 12. It would be suggested here that more weight measurements should be obtained during the aging campaign for more accuracy.

### Paddle-mixer analysis

**Fig. 13:**
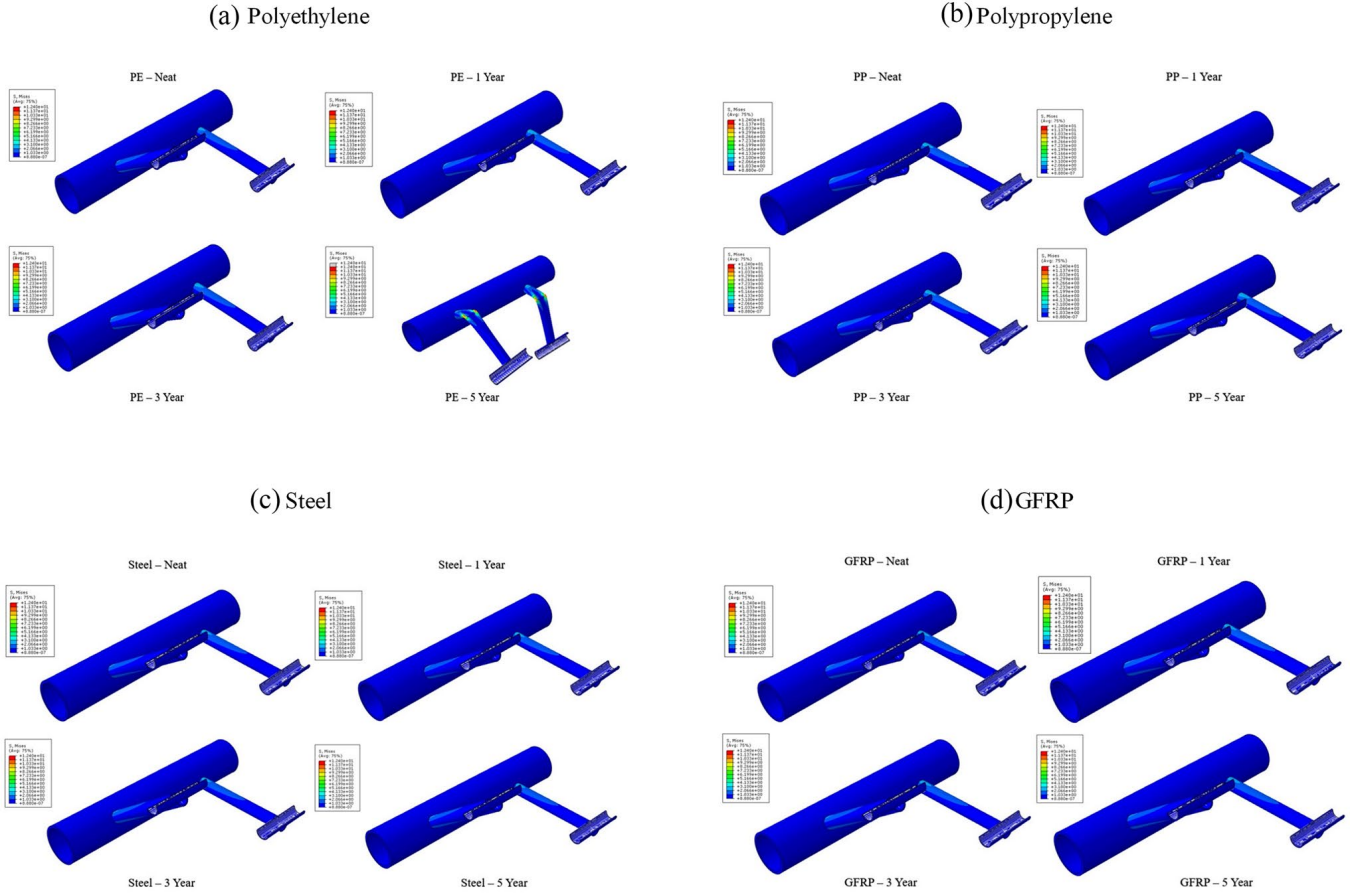
Illustration of Von-Misses stress predictions for all materials for **a** polyethylene, **b** polypropylene, **c** steel and **d** GFRP aging up to 5 years

The minimum and maximum stresses, at the critical points on the impeller, are shown in Table 6. According to the results provided in Fig. 13, the influence of aging was severe in the case of polyethylene. After 3 years, a decrease in the maximum stress by 89% was predicted; therefore, failure occurs after 5 years of aging. On the other hand, polypropylene material presents good mechanical behavior with aging time, since the maximum stress decreases only by 3%. Steel material (St-37) and GFRP remained highly unaffected, with a minor increase of the maximum stress, less than 1.6% and 0.9%, respectively.

**Table 6:** Maximum and minimum stress values as a function of the aging period

	Polyethylene			Polypropylene		
	Minimum stress ${\times 10}^{-5}$(MPa)	Maximum stress (MPa)	Decrease of max stress (%)	Minimum stress $\times {10}^{-5}$(MPa)	Maximum stress (MPa)	Decrease of max stress (%)
Reference	1.45	232	–	1.73	241	–
1 Year	1.26	244	− 5.2	1.32	234	2.9
3 Years	2.15	24.9	89.3	0.97	244	− 1.2
5 Years	1.48	124	46.6	4.24	248	− 2.9

	Steel (St 37)			GFRP		
	Minimum stress ×${10}^{-5}$(MPa)	Maximum stress (MPa)	Decrease of max stress (%)	Minimum stress $\times {10}^{-5}$(MPa)	Maximum stress (MPa)	Decrease of max stress (%)
Reference	0.53	235	–	0.09	233	–
1 Year	0.55	235	0.0	0.14	234	− 0.4
3 Years	0.53	235	0.0	0.11	234	− 0.4
5 Years	0.51	235	0.0	0.11	234	− 0.4

## Conclusions

The main goal of this study was to evaluate long-term strength loss for different construction materials during aging in an anaerobic digester environment. Linear and non-linear regression expressions were used to predict material properties for long-term periods. Ιncreasing the immersion period, stress concentrations severely reduced the allowable yield stress of the materials. After 5 years, the polyethylene paddles are expected to fail, because the maximum stress that could be handled was below the strength of the arms. Steel, GFRP and polypropylene materials remained highly unaffected with a negligible increase of the maximum stress, less than 0.02% and 0.2% and 4% respectively.

It should be highlighted here that although the specimens have different geometry the objective of this study is not comparing the elastic constants nor failure indexes, but the ratio of degradation for each material which is driven by aging mechanisms. Because the material properties are obtained through standards that cannot be neglected, the ratio of degradation of each material is studied in terms of the same paddle-mixer model. Finally, as moisture absorption measurements shown all materials have reached full capacity before 8 months, so this is a good circumstance for comparison.

## Data Availability

Data are available on request from the author.
